# Needs of Chinese patients undergoing home-based rehabilitation after hip replacement: A qualitative study

**DOI:** 10.1371/journal.pone.0220304

**Published:** 2019-07-26

**Authors:** Jing Chen, Xiaoping Zhu, Jinxia Jiang, Yan Qi, Yan Shi

**Affiliations:** Shanghai Tenth People's Hospital, School of Medicine, Tongji University, Shanghai, China; Teesside University, UNITED KINGDOM

## Abstract

**Background:**

The needs of Chinese patients undergoing home-based rehabilitation after hip replacement surgery remain unclear. In this study, we qualitatively investigated the needs of Chinese patients undergoing home-based rehabilitation after hip replacement surgery.

**Methods:**

A total of 21 participants undergoing home-based rehabilitation after hip replacement surgery were included in this study. Individual semi-structured interviews involving all participants were performed to determine the needs and experiences of this patient population during home-based rehabilitation. Data were subjected to qualitative content analysis.

**Results:**

The patients had numerous needs during rehabilitation. Three substantive themes, namely, the need to obtain health-related knowledge, the need to obtain care and support, and the needs of those who cannot perform self-care, were identified from the qualitative data. The participants shared valuable insights into their needs during home-based rehabilitation after hip replacement and provided potential suggestions.

**Conclusions:**

Patients undergoing home-based rehabilitation after hip replacement have several strong needs and lack proper guidance. The initial and continuous engagement of rehabilitation professionals and the establishment of related policies based on patient’s needs are potential approaches for improving the effectiveness of home-based rehabilitation after hip replacement.

## Introduction

China’s elderly population is estimated to reach 243 million by 2020 [1]. In China, the national population has entered the stage of rapid aging, and problems associated with aging will intensify in the next 30 years [2, 3]. The incidence of hip fractures in the elderly in China has increased at an annual rate of 1% to 3% as the national population continues to age [4]. Artificial hip replacement has become one of the most commonly used methods for the treatment of hip disorders and the restoration of hip function in elderly patients [5]. Artificial hip replacement can effectively relieve pain, improve the functional status of limbs, and improve the quality of life of patients [6]. Although surgical treatment can shorten the bed rest period associated with hip-related diseases in elderly patients, long-term rehabilitation determines the extent of the mobility recovery of patients with hip replacement [7]. Most patients receive post hospitalization functional rehabilitation care at home or at community centers. Rehabilitation care after hip surgery covers a wide spectrum of activities and includes hip functional training, daily life activity training, psychological rehabilitation, and related knowledge education [8, 9]. These requirements result in a massive demand for home-based rehabilitation services.

Literature review, however, has shown that the nursing staff for rehabilitation services is insufficient and that patients undergoing home-based rehabilitation receive inadequate guidance. More than 56% of elderly patients who have undergone hip replacement [10–12] have experienced diverse problems, such as persistent dysfunction, joint stiffness, and pulmonary infection due to improper home-based rehabilitation after discharge. Previous study[13] has validated the program theory of the Groningen orthopaedic exit strategy (GOES), a theory-driven program aiming to improve the rehabilitation of total hip and knee arthroplasty patients after shortened hospital stay, and has found that the conceptual theory is supported; however, as the treatment did not influence the mediating variables (action theory), the value was very limited. Furthermore, a recent qualitative study[14] has reported there was a feeling of uncertainty and being left on their own after discharge, and there is a need to develop in partnership with each individual patient a post discharge plan of care and rehabilitation to meet their individual needs, preferences and mode of motivation.

To date, the needs of patients in different stages of home-based rehabilitation after hip replacement surgery remain unclear. We believe that technical support and decision-making based on the characteristics of different stages of home-based rehabilitation are beneficial for ensuring the recovery of patients after hip replacement. Questions surrounding the needs of patients receiving home-based rehabilitation care after undergoing hip replacement surgery, the changes in these needs at various rehabilitation stages, and the type of professional guidance that should be given by healthcare providers to patients undergoing home-based rehabilitation after hip replacement warrant clarification. Thus, we conducted this qualitative study based on interpretative phenomenology analysis (IPA) to understand how a person makes sense of their experience of a particular phenomenon[15], by which to investigate the needs of Chinese patients undergoing home-based rehabilitation after hip replacement, and to provide considerable evidence and insights into these questions.

## Methods

We conducted and reported this study in compliance with the consolidated criteria for reporting qualitative research (COREQ) [16].

### Ethical considerations

Ethical approval was obtained from the Ethics Committee of Shanghai Tenth People’s Hospital.

### Participants and sampling

We identified patients who underwent hip replacement surgery over the period of September 2017 to October 2018 as potential participants. Patients were included if they were aged ≥60 years old and had received hip replacement surgery less than 6 months ago, and underwent home-based rehabilitation immediately after discharge, and able to communicate verbally. We excluded patients who had experienced malignant hip diseases, such as osteomyelitis, malignant tumors, and those who had concurrent diseases that can affect physical activities. Examples of these diseases included cerebrovascular accidents, hemiplegia, and heart failure. We used convenience sampling and all the participants were recruited from one hospital.

### Interviews

Informed consent was obtained from all the participants. We designed semi-structured interviews with open-ended questions to analyze the needs of patients undergoing home-based rehabilitation after hip replacement surgery (Table 1). Interviews were conducted in the time ranges of 1 to 60 days after the operation, and it was conducted in accordance with the principles and guidance of deductive thematic analysis [17]. This approach enabled the in-depth exploration of viewpoints and feedback for home-based rehabilitation. Questions analyzing the possible facilitators and barriers of home-based rehabilitation were adopted. Personal perspectives regarding the setting and design of the rehabilitation program were obtained. In addition, we asked patients to provide suggestions for improving the effects and safety of home-based rehabilitation.

**Table 1:** The guide topics for interviewing.

1	The expectations from the surgery
2	The goals and plans of home-based rehabilitation
3	The experience of rehabilitation at home
4	The disadvantages and advantages of rehabilitation at home
5	The needs of home-based rehabilitation (eg. physical, psychological, financial et al.)
6	The potential solutions to the needs of home-based rehabilitation
7	The suggestions for improving the effects and safety of home-based rehabilitation
8	Other concerns (eg. Consultancy with other people)

Interviews were conducted face-to-face in accordance with the patients’ convenience and for 60–90 min durations. The interviews were conducted by two authors, and all interviews were audiotaped and transcribed verbatim within 3 days after each interview. We also included several valuable nonverbal behaviors, such as silence, thinking, hesitation, pausing, crying, body movements, and facial expressions, observed during the interview.

### Data analysis

All interview transcripts were independently analyzed by two authors by using QSR NVivo 10/11 software. Analysis was conducted on the basis of research questions and participant responses to each question and was grouped to develop the unit of analysis. An iterative process [18] was used to summarize the data descriptively. This process included the deductive coding of relevant passages by using the statements of participants; the organization and grouping of recurring ideas into response categories; and the inductive recoding and condensing of response categories to identify patterns, regularities, and descriptive themes. During the primary analysis, the research members discussed and reviewed the preliminary codes and themes for consistency. We performed the data analysis when the first participant was included, and we stopped recruitment when data saturation was reached; defined as when there were no further new themes or content perceived by two authors independently [19], and any disagreement was resolved through discussion.

## Results

We included 21 participants in this present study. The demographic characteristics of the interviewed participants are presented in Table 2. Eight male and 13 female patients were included. The average age (mean ± SD) of the participants was 66.28 ± 5.21 years old, and living arrangements and accompanying diseases differed among participants.

**Table 2:** The demographic characteristics of interviewed participants.

Participant code	Gender	Age(years)	The waiting times for (total) hip arthroplasty (days)	Hemiarthroplasty or total hip arthroplasty	Duration of hospital stay following surgery(days)	The time after hip-replacement(days)	Living arrangement	Accompanying diseases
1	Female	63	15	total hip arthroplasty	26	31	Living with spouse	Hypertension, diabetes
2	Female	66	62	total hip arthroplasty	16	19	Living with family member	Diabetes
3	Male	63	7	total hip arthroplasty	13	17	Living alone	Hypertension
4	Male	69	42	total hip arthroplasty	15	18	Living with family member	Hypertension
5	Female	74	12	hemiarthroplasty	17	21	Living with spouse	Diabetes, hyperlipidemia
6	Female	61	17	total hip arthroplasty	19	27	Living with spouse	Diabetes
7	Female	66	20	hemiarthroplasty	22	25		
8	Male	69	3	total hip arthroplasty	31	35	Living alone	Hypertension
9	Female	64	48	hemiarthroplasty	39	40	Living alone	Diabetes
10	Male	71	52	total hip arthroplasty	25	27	Living with spouse	Hypertension, diabetes
11	Female	69	3	total hip arthroplasty	27	29	Living with friends	hyperlipidemia
12	Female	73	9	total hip arthroplasty	27	30	Living alone	Hypertension, stroke
13	Female	64	18	total hip arthroplasty	22	24	Living with spouse	Hypertension
14	Male	67	36	hemiarthroplasty	16	19	Living with family member	Hypertension, diabetes
15	Female	62	18	total hip arthroplasty	37	38	Living with spouse	Diabetes
16	Male	64	14	hemiarthroplasty	19	22	Living with family member	Diabetes, hyperlipidemia
17	Female	75	46	total hip arthroplasty	18	20	Living with family member	Hypertension
18	Male	62	60	total hip arthroplasty	21	27	Living with spouse	Diabetes
19	Female	62	3	total hip arthroplasty	33	34	Living with family member	Hyperlipidemia
20	Female	64	11	hemiarthroplasty	24	27	Living alone	Diabetes
21	Male	67	15	total hip arthroplasty	37	42	Living with spouse	Hypertension, hyperlipidemia

The main results (Table 3) of the interviews were obtained after several rounds of analysis and discussion and are as follows:

**Table 3:** The themes and subthemes associated with needs of home-based rehabilitation.

The needs to obtain health-related knowledge
✧ The daily diet knowledge
✧ The diseases-related knowledge
✧ The treatment-related knowledge
✧ The knowledge about the rehabilitation programme
✧ The knowledge about the prognosis
The needs to obtain cares and supports
✧ The prevention and treatment of related complications
✧ The need to facilities renovation
The needs for those who cannot self-care
✧ The needs to obtain guidance from professional medical staff
✧ The needs to be cared by relatives and friends
✧ The needs to obtain social support

### Need to obtain health-related knowledge

#### Daily diet knowledge

Chinese people have a strong demand for nutritional knowledge related to Chinese diet and medicated diet culture. Chinese people switch recipes when they feel unwell and tend to cook various soups or add Chinese medicinal herbs for nourishment after disease or surgery. *Huang Qi*, one of the most commonly used traditional herbal medicine in China, is known as the “best tonic herb.” During the interview, 18 patients mentioned consuming *Huang Qi*.

*“I heard that Huang Qi is good for post-*surgery *recovery*. *Should I eat Huang Qi? What’s the best recipe that uses Huang Qi”* [P16].

In addition, some participants believed that blackfish soup could aid recovery from surgical incisions. Nevertheless, some patients raised doubts about this belief during the interview.

*“Is blackfish soup useful? Should we eat more bone soup to supplement our calcium intake?"* [P8].

#### Disease-related knowledge

Most of the interviewed patients have an inadequate understanding of their disease. Thus, healthcare providers should provide detailed explanations to the patients. In addition, elderly patients generally have reduced cognitive and memory. Thus, they should be repeatedly and carefully informed of essential disease knowledge.

*“I don't know how I acquired this disease*. *I don't remember what you talked about*, *so can you repeat what you had said and give me a paper version of our discussion? I don’t remember the instructions that the doctor and nurse gave me when I was discharged*.*”* [P2].

#### Treatment-related knowledge

Elderly patients believe that the human body cannot withstand surgery and that surgery will injure meridians and cause *Yuan Qi* to disappear. They believe that the disappearance of *Yuan Qi* is harmful to health. Many patients reject surgical options because they believe that surgery can damage vitality. During the interview, patients expressed concern about his or her physical condition and lack of knowledge related to their surgery and treatment plan.

*“Won’t this surgery cause massive injury? How can I address the intense pain caused by the incision? I underwent the surgery as the doctor suggested*, *but I don’t understand what happened*, *and I’m worried about my condition*. *I don't want to know when and where I should get my sutures removed*.*”* [P10].

#### Knowledge about the rehabilitation program

Most patients considered walking after surgery as their most urgent need and pain level as the most worrisome issue in the early postoperative period. However, Chinese culture does not encourage the direct expression of physical and psychological pain, which is different from some other cultures [20]. Moreover, Chinese patients generally resist the use of analgesics. Thus, the contradiction between the aim of medical workers to resolve the patient’s pain and the patient’s resistance to drug use is particularly prominent.

*“I want to relieve my pain*, *but I’m afraid of side-effects of analgesics*. *My pain is intense*, *but my prescription has run out and contacting the doctor is inconvenient*. *I don’t know when and where to get analgesics?”* [P21].

Several participants expressed their concerns about the daily activities related to rehabilitation:

*“Can I climb stairs? When and how can I start climbing stairs again? Can I use auxiliary equipment? Will I recover after half a year? My husband has passed away*, *and I am very worried about whether I can go sweep his tomb during this year’s winter solstice?”* [P3].

#### Knowledge about prognoses

Most patients notice and care about the situation of other patients with the same condition and hope to communicate with each other. They are eager to learn the experience and prognosis of similar cases from healthcare providers or peer patients.

*“I was relieved when I learned that a friend in the other bed in the hospital has been able to walk*. *I hope the doctor can tell how other patients with the same disease are treated and their treatment and rehabilitation prognosis*.*”* [P19].

### Need to obtain care and support

#### Prevention and treatment of related complications

The patients’ fear of complications, such as dislocation, warrants the knowledge of prevention strategies. Patients prevent feeling pain caused by excessive hip-joint flexion by avoiding kneeling or bending over. The need to avoid certain movements seriously affects the quality of life of the patients. Healthcare workers should provide personalized instruction manuals and educational guidance. However, the awareness of the adverse effects of several other complications and overall awareness of the rehabilitation program are lacking.

*“I was scared when the doctor said that we should not do things like this or that*. *I was afraid to come back to the hospital for a second operation*, *and I don’t dare to go out alone*. *Can you tell me the specific things that I should and should not do? Do other complications exist? I do not understand them*. *Although nurses and doctors told me that I should move often*, *I do not dare to move and prefer to just stay in bed*.*”* [P2].

#### Need to renovate facilities

Seventeen families did not modify the family residence to meet the patient’s rehabilitation needs. For example, they did not provide bed rails for bedridden patients, install armrests in the bathroom, or replace chairs with low heights and arms. These actions are not conducive for the functional rehabilitation of elderly patients. Currently, no professional companies and personnel can install relevant equipment and home facilities for the safety of elderly patients, and comprehensively transforming home facilities is difficult for patients and their families. Most of the elderly patients interviewed expressed demand and distress in this regard.

*“The doctor said that I have to sit a little higher*, *but how high is it? I don't know where to get a toilet as such; facilities renovation takes a lot of money*, *I just do the rehabilitation temporary*, *it doesn't take long*, *renovate the home isn’t necessary*.*”* [P15].

### Needs of those who cannot perform self-care

#### Need to obtain guidance from professional medical staff

Most patients have to go through a period of partial self-care during rehabilitation. Although patients depend on and trust professional healthcare providers, they want special care and guidance from doctors and nurses.

*“I prefer that the doctor calls to ask me about my rehabilitation progress and tell me what to do*. *Of course*, *I hope that the doctor and nurse contact me*, *but I feel embarrassed to call the doctor because I know that they’re busy*. *I do hope that they can provide me with constant guidance*.*”* [P16].

In addition to seeking medical care from institutions, patients prefer to seek help from large public hospitals rather than community centers based on previous medical experience.

*“Of course I hope that I will still be treated at a large hospital*. *I don't want to go to a community hospital or a rehabilitation institution*. *I prefer guidance from a doctor who had performed surgery at a large hospital*.*”* [P4].

#### Need to be cared for by relatives and friends

The family is the most important component of patient care in the Chinese cultural context. Family members are obligated to take care of the patients during hospitalization or after discharge. Otherwise, they will face pressure from public opinion. Friends go on hospital visits to care for patients.

*“Of course*, *I hope my daughters will take care of me but they are busy*, *and my son-in-law is away on a business trip*. *I am worried because my son cannot take care of me*. *My sister took care of me during my recovery*, *and I am happy and recovering well*.*”* [P18].

#### Need to obtain social support

Most elderly patients have reduced self-care ability after hip joint replacement, and family members are usually required to undertake a number of tasks, such as hospital transport, registration, and visits. These tasks are associated with a heavy social burden. The societal needs and appeals of elderly patients include additional support, such as improved nursing care after discharge. Some patients stated that they did not understand community services and functions.

*“I still have high blood pressure*. *I have to go to the hospital every two weeks to get medicine*. *Doing so is inconvenient for me now*. *I cannot travel a long way*. *Taking me to see a doctor is difficult for my children*. *I do not know any policies or institutions that can help us*.*” “I do not know what services are available in community hospitals*.*”* [P7].

Patients are concerned with their connection to society and the changes in their social roles. They are unable to maintain normal social interaction because their social networking capabilities are limited by their physical impairments.

*“I used to ride electric motors with a motor team*. *However*, *I can only stay at home now*, *and my team members think about me*. *I used to perform housework*, *and now the family residence is a mess*.*”* [P9].

## Discussion

Elderly patients have declining physiological functions, increased incidence of chronic diseases, and reduced quality of life [21]. Elderly patients who have experienced sudden fractures lack disease knowledge and find coping with diseases difficult [22]. Their health is seriously threatened. Thus, supports from family and professional healthcare providers are essential for improving the prognosis of patients who had undergone hip replacement surgery [23, 24]. It’s been reported[25] that the common injury mechanism of acetabular fracture in elderly is related to fall injury, and in this study, 19 patients underwent the hip replacement with regards to the fall injury, only 2 patients underwent the surgery because the car accidents. At present, most elderly patients require home-based rehabilitation, and the family acts as the main caregiver. Improving the home care and guidance of elderly patients is an issue that should be considered. In this study, we investigated the needs of elderly patients undergoing home-based rehabilitation after hip replacement surgery to determine the demands of elderly care services and to provide a basis for the establishment of home care or service centers.

The guidance received by patients during hospitalization or home-based rehabilitation care should be strengthened, and ensuring that patients adhere to rehabilitation plans and form good rehabilitation exercise habits is beneficial for improving the prognosis of patients with hip replacements [26, 27]. The pertinence and effectiveness of health education should also be strengthened [28]. Although patients who have received hip replacements can obtain rehabilitation knowledge from various aspects and multiple channels, their rehabilitation knowledge is generally incomplete or fragmentary, and they may sometimes even be misinformed because systematic rehabilitation knowledge education remains lacking [29]. Thus, healthcare providers should develop individualized health education plans for the long-term rehabilitation of patients with hip replacements even before their discharge from the hospital [30]. The rehabilitation plan should be deployed beginning with patient’s admission to home-based rehabilitation; moreover, adequate evaluation and supervision are required to ensure the effectiveness of rehabilitation [31, 32].

Our results indicate that establishing a community-based rehabilitation support system is necessary. Most interviewed patients expressed their concerns regarding their lack of access to related knowledge, such as pain control and detailed rehabilitation movement [33, 34]. The current demand of patients for community services reflects the gaps and weaknesses in community rehabilitation care. Previous studies [35, 36] indicated that the costs of home-based rehabilitation are lower than those of hospital-based rehabilitation. Thus, maximizing the effect of community support on patients with hip replacements and providing patients with comprehensive guidance should be the focus of existing studies, and corresponding policies should be established to actively promote the construction of community and private rehabilitation institutions. Encouraging large hospitals to support and guide small and medium-sized community hospitals may also help [37]. Family facility and environment assessment and renovation should be supported by social and professional institutions to improve the safety and efficacy of home-based rehabilitation for elderly patients [38, 39].

Adequate information supports rehabilitation, and personalized guidance and information on the prevention of related complications should be provided to patients. In particular, rehabilitation activities tailored to different patients and delivered through different programs are required, and timely follow-up and contact with patients and their families and friends are warranted [27]. Teaching resources for elderly patients are limited given the unfamiliarity of this patient group with the use of mobile phones or computers [40]. At present, medical information and technology are combined, and elderly patients require additional attention from healthcare providers given their status as a vulnerable group. Paper-based instruction manuals for health education with large print are highly convenient and widely accepted by elderly patients [41, 42]. Meanwhile, rehabilitation videos and other information can be delivered through mobile technology to patients and their families who are skilled in using mobile devices, such as smartphones [22, 43].

This study has several limitations that must be acknowledged. First, we recruited patients from only one hospital, the services from different hospitals and areas can produce some influences on the needs of patients undergoing rehabilitation after hip replacement. Future studies in a broader area ranges are needed. Second, the included participants were highly cooperative. Therefore, their experience may not reflect the experience of the typical elderly adult undergoing rehabilitation after hip replacement. However, similarities have been found between the themes identified in our study and those identified in other studies [31, 44] among different countries based on qualitative methods. Nevertheless, our study has several strengths given that it is the first study that focused on the needs of patients undergoing home-based rehabilitation after hip replacement in China. Thus, this present study provides evidence and insights for clinical applications and future policy-making.

## Conclusions

Numerous needs have been identified for the patients undergoing home-based rehabilitation after hip replacement surgery. Although most of these patients showed a strong desire for recovery, they lacked proper rehabilitation guidance. In addition, we has found that these patients have a massive demand for professional home-based rehabilitation services. Policies involving community and home visits by healthcare providers to provide professional guidance are necessary to improve the management of the rehabilitation care of patients with hip replacements.
